# Supplementary material for: Myosin-VIIa is expressed in multiple isoforms and essential for tensioning the hair cell mechanotransduction complex
Source: Nat Commun. 2020 Apr 29;11:2066. doi: 10.1038/s41467-020-15936-z (PMC7190839; doi:10.1038/s41467-020-15936-z)
Supplement: Supplementary file 1 — Supplementary Information [file 41467_2020_15936_MOESM1_ESM.pdf]

Supplementary Information

Li et al.,

Myosin-VIIa is expressed in multiple isoforms and essential for tensioning the hair cell mechanotransduction complex

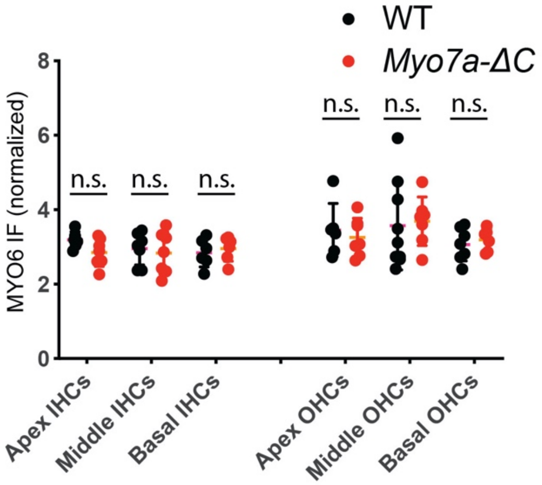

Supplementary Figure 1. MYO6 levels are not affected in *Myo7a-ΔC* cochlear hair cells:
Organ of Corti from P6-7 WT and *Myo7a-ΔC* mice were stained with the anti-MYO6 antibody (Santa Cruz). MYO6 fluorescence intensity in a volume encompassing the cuticular plate and the cytosol overlying the nuclei was measured using grey value analysis in ImageJ and normalized to non-specific staining in the inner sulcus region. Per organ and animal, 30 OHCs and 10 IHCs were analyzed. n (number of animals) for apical (5), middle (6) and basal(5) IHCs in WT. n (number of animals) for apical (5), middle (8) and basal(6) OHCs in WT mice. n (number of animals) for apical (7), middle (7) and basal(6) IHCs in *Myo7a-ΔC* mice. n (number of animals) for apical (7), middle (7) and basal(6) OHCs in *Myo7a-ΔC* mice. Data are plotted as mean +/- SD, and n.s. statements derive from a 2-way ANOVA test, followed by a Sidak's multiple comparion test. Source data are provided in the Source Data file.

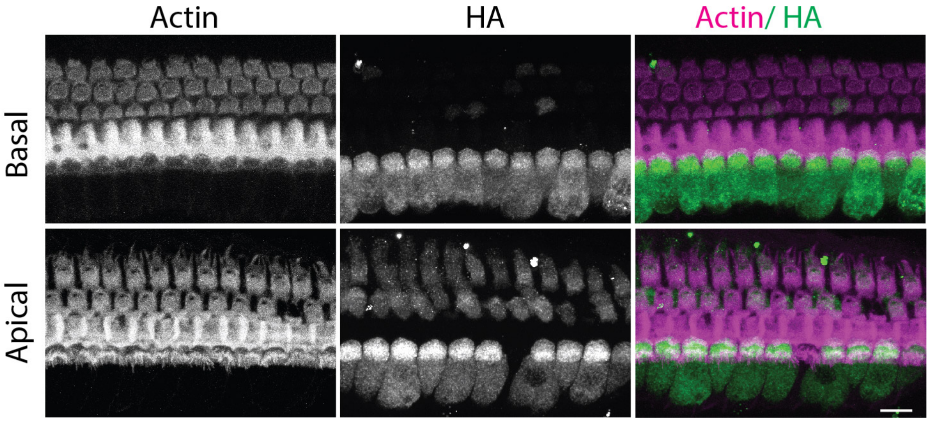

Supplementary Figure 2. HA immunoreactivity in apical and basal cochlear hair cells of mature (P30) *HA-Myo7a-C* KI mice. HA-MYO7A-C expression is comparable to the expression pattern observed at P6 (**Fig. 3d**) (scale bar: 10 µm).

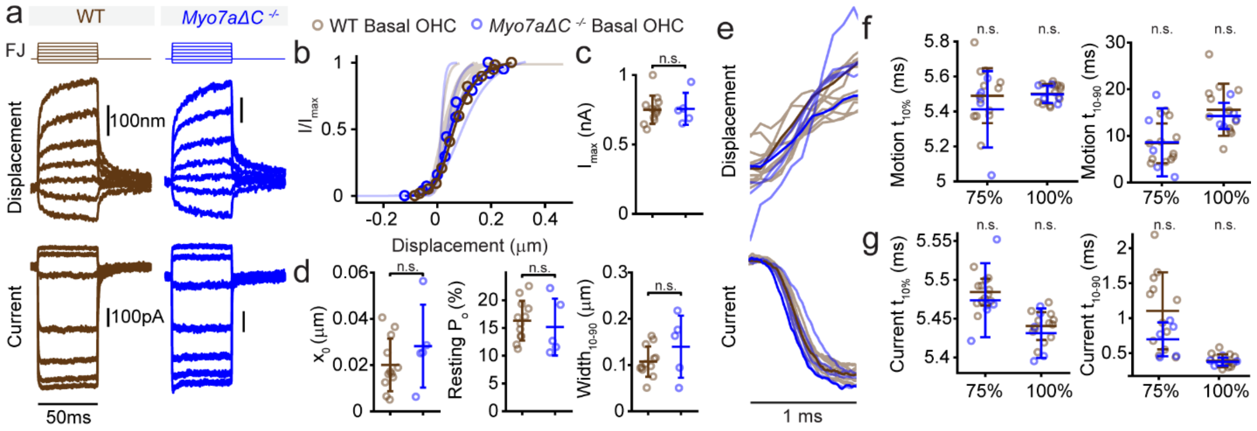

Supplementary Figure 3. Basal *Myo7a*-ΔC OHCs do not exhibit changes in the activation curves or the current onset kinetics that were observed in IHCs. **a.** Displacement and current traces elicited by step-like force stimulation of basal turn OHCs from WT (brown) and *Myo7a*-ΔC (blue) mice. FJ indicates the voltage waveform delivered to the fluid jet. **b**. Activation curves show no difference. Light colored traces show all the activation curve fits of all cells recorded. **c**. No effect was seen in the peak current. **d**. No effect was observed in the position of the activation curve (x_0), the resting P_o, or the width of the activation curve ($Width_{10-90}$). **e.** When zooming in on the onset (1 ms timeframe), there was no difference in the initial rise of the hair bundle displacement or the current onset. The step eliciting ~75% peak currents are shown for all cells with the data from panel **a** shown with darker colors. The motion traces are significantly more noisy due to the smaller displacements elicited in OHCs. **f**. Analysis of the kinetics of the motion and **g.** the current showed no differences. Data for the motion at 50% peak current was omitted because of the low signal to noise ratio due to the smaller displacements for OHCs and led to problems with the automated analysis. For panel c-f, summary plots are represented as mean±SD with WT: n = 12 cells from 7 animals, *Myo7a*-ΔC: n = 5 cells, 4 animals; all statistics presented in the figure derive from unpaired, two-tailed t-tests. Source data are provided in the Source Data file.

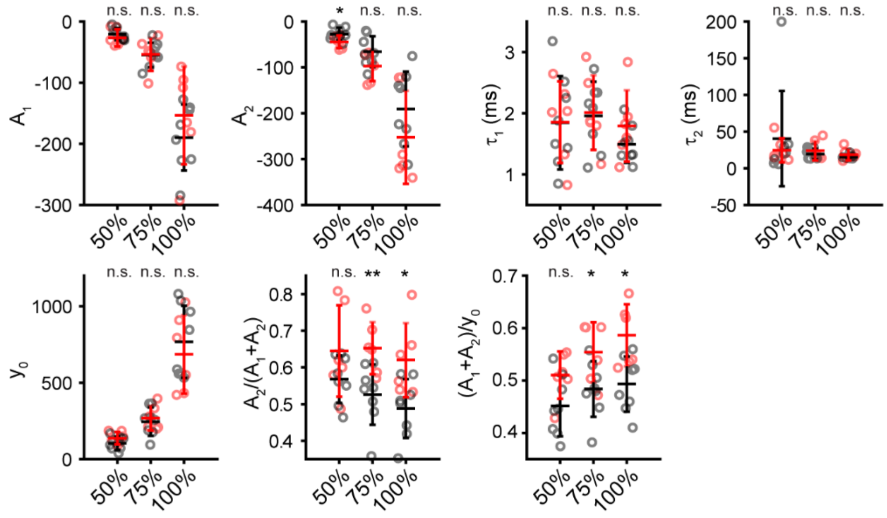

Supplementary Figure 4. Analysis of the creep kinetics for WT (black) and *Myo7a-ΔC* (red) IHCs. The creep of the hair bundle was fit a double exponential equation. A_1, A_2, τ_1, τ_2, and y_0, are parameters directly from the fitting. $A_2/(A_1+A_2)$ was calculated from these parameters and quantifies the relative contribution of the slow creep component. $(A_1+A_2)/y0$ also is calculated from the fit parameters and quantifies the relative magnitude of the creep as compared to the total motion. These parameters are shown for step sizes eliciting about 50%, 75%, and 100% peak current. These data indicate that the slower creep component is the greater in *Myo7a*-ΔC mice. All plots are represented as mean±SD with WT: n = 8 cells from 7 animals, *Myo7a-ΔC*: n = 6 cells from 6 animals; all statistics presented in the figure derive from unpaired, two-tailed t-tests. Source data are provided in the Source Data file.

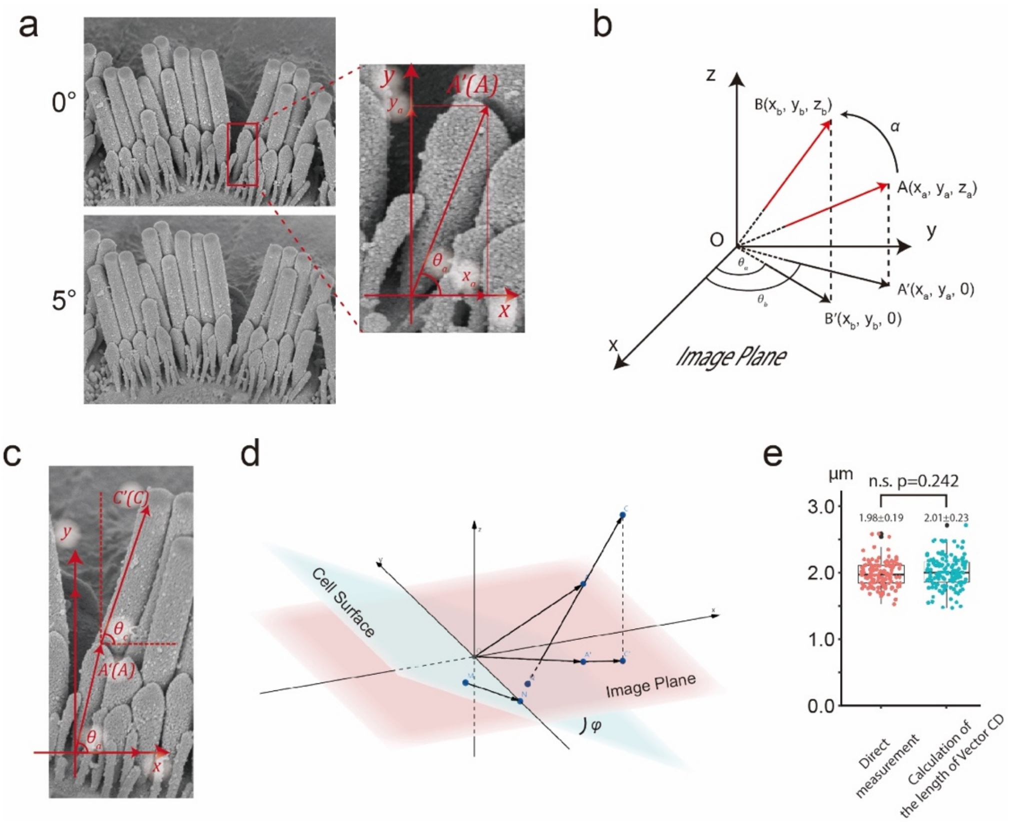

Supplementary Figure 5:

General principle:
SEM stereo-images provide two different imaging planes with a known angle between them. This allows the reconstruction of the three-dimensional coordinates of all visible points (stereocilia tips and insertion points) using basic linear algebra. This in turn is used to calculate the actual lengths of the stereocilia employing Euclidean metric in three dimensions. For calculating the lengths of stereocilia with obstructed stereocilia bases, we use one image and the angle between the oxy plane and the cell surface (assuming that locally it is close to a plane). We continue the vector between two visible points of the stereocilia, and find its intersection with the cell surface using basic linear algebra. Using this calculated invisible point, we find the length using Euclidean metric in three dimensions.

Detailed description:
Calculation of the length of fully visible stereocilia, tilted in any direction (Illustrated in **a** and **b**):
a. A stereo-pair of SEM images was taken with a tilt of α degrees (5 degree in the image pair). For optimal application of this method, eucentric stereo-pairs of hair cells should be taken (hair bundle base lies parallel to the rotation axis of the SEM stage). With this assumption in place, we can measure each stereocilium as a vector. In the model described in **b**, vector OA represents a stereocilium, and vector OB the same stereocilium tilted at an angle α. Their corresponding projections onto the image plane are OA' and OB' (the side edge of the SEM image corresponds to the y-axis), respectively. The lengths of these projections P_a and P_b (length of the stereocilia as they project onto the image plane), and the angles θ_a and θ_b (angles between vectors A' and B' and the x-axis) were measured in ImageJ. The coordinates of vector A' and B' were then calculated using the equations below:

$$\begin{cases} x_a = P_a \cdot \cos\theta_a \\ y_a = P_a \cdot \sin\theta_a \end{cases}$$
$$\begin{cases} x_b = P_b \cdot \cos\theta_b \\ y_b = P_b \cdot \sin\theta_b \end{cases}$$

By using the coordinates of A' and B', the length of the stereocilia can be calculated using the formula below:

$$\delta = \cos \alpha$$

$$Stereocilia\ Length = \sqrt[2]{\frac{x_a^2 + x_b^2 + y_a^2 + y_b^2 - 2\delta x_a x_b - 2\delta y_a y_b + \sqrt{-4(-1+\delta^2)(x_a x_b - \delta(x_a^2 + y_a^2) + y_a y_b + ((-1+2\delta^2)x_a^2 + x_b^2 - y_a^2 + y_b^2 + 2\delta^2 y_a^2 - 2\delta x_a x_b - 2\delta y_a y_b)^2}}{2(-1+\delta^2)}}$$

Calculation of the length of stereocilia with obstructed bases (Illustrated in **c** and **d**):
Five parameters are needed: (1) the angle of the shorter row stereocilium (θ_a) to the x-axis, (2) the length of the projection of the shorter row stereocilium (P_a), (3) the angle of the longer row stereocilium (θ_c) to the x-axis, and (4) the length of the projection of the step of the longer row stereocilium (P_c), defined as the distance between the tip of the longer row stereocilium and the tip of the shorter row stereocilium in front of it (segment AC and A'C')), (5) the angle between cell surface and image plane. Using the method shown above, the length of the stereocilium and the step can be also calculated (L_a, L_c). The coordinates of the tips of the shorter and longer row stereocilia (Point A and Point C in the figure D) can then be calculated using the formulae below:

$$\begin{cases} x_a = P_a \cdot \cos\theta_a \\ y_a = P_a \cdot \sin\theta_a \\ z_a = \sqrt{L_a^2 - P_a^2} \end{cases}$$

$$\begin{cases} x_c = P_c \cdot \cos\theta_c + x_a \\ y_c = P_c \cdot \sin\theta_c + y_a \\ z_c = \sqrt{L_c^2 - P_c^2} + z_a \end{cases}$$

To calculate the angle between the cell surface and the image plane (angle φ), two landmarks on the cell surface are selected (point M and point N). The vector MN should be perpendicular to the x-axis. The length of the projection of MN can be measured using ImageJ. Then, the same points are selected on the stereo-pair of the SEM. Using the same method as used to determine the length of the shorter row stereocilia, the length of the vector MN can be calculated. And the angle φ can be calculated by using the equation below:

$$\varphi = \arccos\left(Projection_{MN}/Length_{MN}\right)$$

With angle φ known, the coordinate of the point Q, which represents the (invisible) root of the longer row stereocilium, will be calculated as the intersection between vector AC and the cell surface.

$$\begin{cases} x_q = \dfrac{x_a y_c \tan\varphi - x_c y_a \tan\varphi - x_c z_a + x_a z_c}{y_c \tan\varphi - y_a \tan\varphi - z_a + z_c} \\ y_q = \dfrac{-y_c z_a + y_c z_a}{(y_c - y_a)\tan\varphi - z_a + z_c} \\ z_q = y_q \tan\varphi \end{cases}$$

After calculating the coordinates of Q, the length of vector CQ, representing the length of the longer row stereocilium, can be calculated using the equation below:

$$Stereocilia\ length = \sqrt{(x_q - x_c)^2 + (y_q - y_c)^2 + (z_q - z_c)^2}$$

The calculations were performed in R.

e. **Validation:** To validate the method described in ***c*** and ***d***, we compared the lengths of the longest row of stereocilia in 6-week old apical OHC stereocilia as determined by two different methods: For stereocilia with visible stereocilia bases, we directly calculated their length by using the method described in panel ***a*** and ***b*.** For the stereocilia with invisible bases, we used the method described in panel ***c*** and ***d***. T-test indicated no significant difference between these two methods (# of stereocilia: directly measurement=129, vector length calculation=139).
