# Supplementary material for: Myosin-VIIa is expressed in multiple isoforms and essential for tensioning the hair cell mechanotransduction complex
Source: Nat Commun. 2020 Apr 29;11:2066. doi: 10.1038/s41467-020-15936-z (PMC7190839; doi:10.1038/s41467-020-15936-z)
Supplement: Supplementary file 3 — Reporting Summary [file 41467_2020_15936_MOESM3_ESM.pdf]

nature research

Corresponding author(s): Jung-Bum Shin
Last updated by author(s): Feb 28, 2020

Reporting Summary

Nature Research wishes to improve the reproducibility of the work that we publish. This form provides structure for consistency and transparency in reporting. For further information on Nature Research policies, see Authors & Referees and the Editorial Policy Checklist.

Statistics

For all statistical analyses, confirm that the following items are present in the figure legend, table legend, main text, or Methods section.

n/a	Confirmed	
☐	☒	The exact sample size (*n*) for each experimental group/condition, given as a discrete number and unit of measurement
☐	☒	A statement on whether measurements were taken from distinct samples or whether the same sample was measured repeatedly
☐	☒	The statistical test(s) used AND whether they are one- or two-sided<br>*Only common tests should be described solely by name; describe more complex techniques in the Methods section.*
☐	☒	A description of all covariates tested
☒	☐	A description of any assumptions or corrections, such as tests of normality and adjustment for multiple comparisons
☐	☒	A full description of the statistical parameters including central tendency (e.g. means) or other basic estimates (e.g. regression coefficient) AND variation (e.g. standard deviation) or associated estimates of uncertainty (e.g. confidence intervals)
☐	☒	For null hypothesis testing, the test statistic (e.g. *F*, *t*, *r*) with confidence intervals, effect sizes, degrees of freedom and *P* value noted<br>*Give P values as exact values whenever suitable.*
☒	☐	For Bayesian analysis, information on the choice of priors and Markov chain Monte Carlo settings
☒	☐	For hierarchical and complex designs, identification of the appropriate level for tests and full reporting of outcomes
☒	☐	Estimates of effect sizes (e.g. Cohen's *d*, Pearson's *r*), indicating how they were calculated

Our web collection on statistics for biologists contains articles on many of the points above.

Software and code

Policy information about availability of computer code

Data collection	Image data collection was performed using the Zen software (vers 2.3, Zeiss) or managed through NIS-Elements software (vers 4.5, Nikon Instruments). For ABRs, the SmartEP (vers. 5.41.02) and for DPOAEs, the SmartOAE software (vers. 5.40.01) were used to collect data (Intelligent Hearing Systems).
Data analysis	Analysis of confocal images were performed using ImageJ (vers. 2.0.0), NIS-Elements software (vers. 4.5, Nikon) or Imaris (vers/ 8.1.2, Bitplane).<br>Graph Pad Prism Vers. 7 and R (vers. 1.2.5019) for statistical analysis. Figures were generated using Adobe Illustrator Vers. 23.0.3<br>For data obtained from patch clamp electrophysiology experiments, quantification and statistical analysis were analyzed using jClamp (vers. 30.5, SciSoft), Matlab (vers. 9.1MathWorks), Excel (vers. 16.16.19, Microsoft), and Prism (vers. 7, GraphPad). CRISPR targets were selected using the CRISPOR online tool (vers. 4.2 and higher).

For manuscripts utilizing custom algorithms or software that are central to the research but not yet described in published literature, software must be made available to editors/reviewers. We strongly encourage code deposition in a community repository (e.g. GitHub). See the Nature Research guidelines for submitting code & software for further information.

Data

Policy information about availability of data

All manuscripts must include a data availability statement. This statement should provide the following information, where applicable:

- Accession codes, unique identifiers, or web links for publicly available datasets
- A list of figures that have associated raw data
- A description of any restrictions on data availability

All data described in the manuscript are available upon request. Raw data are provided in the Source Data file.

Field-specific reporting

Please select the one below that is the best fit for your research. If you are not sure, read the appropriate sections before making your selection.

☒ Life sciences ☐ Behavioural & social sciences ☐ Ecological, evolutionary & environmental sciences

For a reference copy of the document with all sections, see nature.com/documents/nr-reporting-summary-flat.pdf

Life sciences study design

All studies must disclose on these points even when the disclosure is negative.

Sample size	For hearing tests, historical variance of ABR thresholds in the Shin lab was used for the power analysis, with an expectation of a P-value of 0.05. For patch-clamp recording, historical lab data with changes in MET properties informed our power analysis to determine the minimum number of n. For all other experiments, pilot experiments were performed to estimate variance, and a power analysis was performed to estimate sample size.
Data exclusions	no data were excluded
Replication	All findings presented in the present study either underwent rigorous statistical treatment (involving multiple independent replications) or in case of qualitative statements (e.g. differences reported in stereocilia tip prolation in scanning electron microscopy studies), findings were replicated multiple times. All replication attempts gave consistent results.
Randomization	Randomization for mouse work was achieved by distributing experimental groups (Myo7a-ΔC or WT) across cages (in other words, cages did not indicate experimental group).
Blinding	Data was collected and analyzed in a blinded fashion for most quantifications described in the manuscript except the physiology experiments: ABR functional hearing tests: For the longitudinal hearing tests that were performed over a course of 9 weeks, the experimenter often inadvertently "memorized" tag numbers and their corresponding genotypes, despite initial blinding, because data was analyzed at each age the hearing performance was assessed. To prevent bias, however, analysis of the data (threshold determination based on recorded traces) was confirmed by an independent experimenter.<br>Patch clamp electrophysiology experiments: The selection of the mice was not blinded (the experimenter knew the genotype when performing the patch clamp recording. However, the data collection occurred over multiple days with mutant and WT interspersed. Importantly, the analysis was automated to remove bias.

Reporting for specific materials, systems and methods

We require information from authors about some types of materials, experimental systems and methods used in many studies. Here, indicate whether each material, system or method listed is relevant to your study. If you are not sure if a list item applies to your research, read the appropriate section before selecting a response.

Materials & experimental systems

n/a	Involved in the study	
☐	☒	Antibodies
☒	☐	Eukaryotic cell lines
☒	☐	Palaeontology
☐	☒	Animals and other organisms
☒	☐	Human research participants
☒	☐	Clinical data

Methods

n/a	Involved in the study	
☒	☐	ChIP-seq
☒	☐	Flow cytometry
☒	☐	MRI-based neuroimaging

Antibodies

Antibodies used	Rabbit polyclonal Myosin-VIIa antibody (catalog#: 25-6790, Proteus BioSciences Inc, Ramona, CA. 1:100), mouse monoclonal Myosin-VIIa antibody (Developmental Studies Hybridoma Bank, MYO7A clone 138-1, concentrate, 1:100), mouse monoclonal Myosin-VI antibody (A-9, Santa Cruz, 1:100), rabbit anti-Harmonin antibody (H3, obtained from Ulrich Mueller lab), rabbit anti-ADGRV1 antibody (obtained from Dominic Cosgrove's lab), rabbit polyclonal Myosin-VIIa antibody used in Fig. 2 (PB206) was custom-generated and affinity purified against the immunizing MYO7A peptide LPGQEGQAPSGFEDLERGR
Validation	All antibodies for Myosin-VIIa and Myosin VI were validated by confirming absence of immunoreactivity in KO mice. The anti-harmonin antibody was validated by confirming relocalization of harmonin staining in Myo7a KO mice. The anti-ADGRV1 antibody from the Cosgrove lab was validated in KO mice (McGee et al. 2006. The very large G-protein-coupled receptor VLGR1: a component of the ankle link complex required for the normal development of auditory hair bundles)

Animals and other organisms

Policy information about studies involving animals; ARRIVE guidelines recommended for reporting animal research

Laboratory animals	This study involved the use of the following strains of mice:<br>- Myo7a-ΔC mouse (maintained on C57Bl6/J background). Either sex was used for experiments. Experiments were performed at ages P5-P9, P17, 4 weeks, 6 weeks, 9 weeks.<br>- Myo7a- full KO mouse (SJL/C57Bl6/J mixed background). Either sex was used for experiments. Experiments were performed at ages P5.<br>- HA-Myo7a-C KI mouse (SJL/C57Bl6/J mixed background). Either sex was used for experiments. Experiments were performed at ages P5.<br>- Myo7a::beta-Actin-GFP transgenic mouse (maintained on C57Bl6/J background). Either sex was used for experiments. Experiments were performed at ages P6.
Wild animals	we did not use wild animals
Field-collected samples	we did not collect field samples
Ethics oversight	The protocol for care and use of animals was approved by the University of Virginia Animal Care and Use Committee. The University of Virginia is accredited by the American Association for the Accreditation of Laboratory Animal Care

Note that full information on the approval of the study protocol must also be provided in the manuscript.
